# Parts, Period, and Extraction Solvents as Parameters Influencing *Harungana madagascariensis* Lam. ex Poir. (Hypericaceae) Antibacterial Activity

**DOI:** 10.1155/2021/6615596

**Published:** 2021-03-11

**Authors:** D. Deutou Tégaboué, C. L. Cidjeu Pouaha, R. M. Ebelle Etame, Sikam K. G., D. Ngo Hagbe, Issa Moussa, Raphael Tchientcheu, R. S. Mouokeu, R. A. Ngono Ngane

**Affiliations:** ^1^Laboratory of Biochemistry, Department of Biochemistry, Faculty of Sciences, University of Douala, P.O. Box 24157, Douala, Cameroon; ^2^Institute of Medical Research and Medicinal Plant Studies (IMPM), P.O. Box 13033, Douala, Cameroon; ^3^Laboratory of Chemistry, Department of Chemistry, Faculty of Sciences, University of Douala, Douala, Cameroon; ^4^Laboratory of Ecosystems and Fisheries Resources, Institute of Fisheries and Aquatic Sciences, University of Douala, P.O. Box 7236, Douala, Cameroon

## Abstract

The study of the conditions which affect the pharmacological activity of medicinal plants is the basis for the development of phytomedicine. This study aimed at identifying some optimal conditions for antibacterial activity of extracts of *Harungana madagascariensis*. The leaves, bark, and roots were harvested in Douala, Littoral Region of Cameroon, in August 2019 at three times of the day: early in the morning (6 a.m.), at midday (12 p.m.), and in the afternoon (6 p.m.). They were dried at room temperature and ground to give a fine powder. Each powder was macerated in methanol for 72 h and boiled in distilled water and palm wine. The obtained extracts were evaluated for their antibacterial activity by broth microdilution method on 31 clinical and 4 reference bacterial strains. The results show that the best extraction yield was obtained using the bark extracted from palm wine by decoction. The methanol bark extract was found to be more active than other solvents (8 and 512 *µ*g/mL). In fact, the activity of the 6 a.m. extract was significant on 4 of the 35 strains tested (MIC< 100 *µ*g/mL) and moderate on 27 of the 35 strains tested (100≤ MIC≤ 625 *μ*g/mL). The activity of the noon extract was significant on 8 strains and moderate on 23 strains and, finally, the activity of the extract of 6 p.m. was significant on 9 strains and moderate on 25 strains. The extract harvested at 6 p.m. inhibited the growth of the 35 strains tested and revealed significant activity (MIC <100 *μ*g/mL) on *Staphylococcus aureus*, *Escherichia coli*, *Citrobacter freundii*, *Serratia marcescens*, *Salmonella enterica* serovar *typhi*, and *Yersinia enterocolitica*, making this hour the best harvesting time. This study further confirms that the bark of *H. madagascariensis* is a potent antibacterial agent, being better still when harvested at 6 p.m. and extracted with methanol.

## 1. Introduction

Infectious diseases are one of the leading causes of death worldwide [1]. According to the World Health Organisation, these diseases are responsible for 17 million deaths per year worldwide [2]. To overcome these infectious diseases, many antibacterials are available, including tetracycline, amoxicillin, amoxicillin + clavulanic acid, doxycycline cotrimoxazol. penicillin, imipenem, and amikacin. However, probabilistic prescriptions and the nonrational use of antibiotics have led to the development of bacterial resistance. In addition, the high cost of these antibiotics, the duration of the treatment, and poverty accentuate this phenomenon in developing countries. These justify the constant search for antimicrobial substances. The use of medicinal plants is an alternative, due to their accessibility, availability, and great diversity in bioactive molecules [3]. Indeed, the antimicrobial properties of many plants have been investigated and they are also used extensively in daily clinical practice. Their active principle have led to the development of some modern available medicine such as aspirin, digitoxin, morphine, quinine, and pilocarpine [4].

Cameroon, Africa in miniature, is home to a set of important and varying plant species, including *H. madagascariensis* commonly called Nketto by the Bamileke tribe. It is a tropical plant, abundant in the savana and the forest region [5]. Its antibacterial and antifungal properties have been established [6]. Many studies reveal the antibacterial properties of different parts of this plant, extracted with several solvents, including methanol, water and palm wine. The antibacterial activity of the leaf extract was established by Moulari et al., [6] and Tankeo et al., [7]. The antibacterial properties of methanol extract from stem bark have been highlighted by Hopogap [8] and Ebelle Etame et al. [9]. In addition, works on the antibacterial activity of methanol extracts, fractions, and compounds of *H. madagascariensis* were investigated [7]. This antibacterial activity was associated with the presence of betulinic acid, ferruginin, and kaempferol-3-0-*β*- D-glucopyranoside [7]. Following the work of Magne [10] on the influence of parts and the harvest period on the antifungal activity of *H. madagascariensis*, it was clearly established that the roots of this plant harvested at noon had the best activity. Based on Jaradat et al.'s work [11], it was found that the geographical location and ecological conditions affect the chemical constituents and the antibacterial activity of the essential oil of *Ruta chalepensis* leaves in Palestine. It, therefore, appears that the antimicrobial properties of *H. madagascariensis* are well documented. However, plant antimicrobial activity may vary according to the part [10], extraction solvent [5], harvest period [10], and harvest location [12]. With regard to the antibacterial activity of *H. madagascariensis*, it seems important to seek the best conditions for the expression of the antibacterial activity. It is in this context that this work falls, the objective of which is to identify the optimal conditions for antibacterial activity of extracts of *H. madagascariensis*, more specifically, (1) to assess the influence of the plant's part on the yield and antibacterial activity of *H. madagascariensis*, (2) to determine the effect of the harvest time on the yield and antibacterial activity of *H. madagascariensis*, (3) and to assess the effect of the extraction solvent on the yield and antibacterial activity of *H. madagascariensis*.

## 2. Materials and Methods

### 2.1. Materials

#### 2.1.1. Plant Materials

The parts used in this study consisted of the leaves, barks, and roots of *H. madagascariensis*. They were harvested in Douala in the Littoral Region (Cameroon), more precisely at PK17. The different parts were harvested in August 2019 at three times of the day: early in the morning (6 a.m.), at midday (12 p.m.), and in the afternoon (6 p.m.). The identification of the plant was confirmed at the National Herbarium of Cameroon by comparison with the reference sample N^o^ 4224 HNC.

#### 2.1.2. Microorganisms

31 clinical strains and 4 reference strains of enteric bacteria were used in the study. Clinical strains were isolated from stool of patients with gastrointestinal problems. The reference strains were a kind gift of Professor Kuete Victor, Department of Biochemistry, University of Dschang, Cameroon. The sensibility of all the strains to commonly used antibiotics is summarised in Table 1.

### 2.2. Methods

#### 2.2.1. Extract Preparation

The bark and roots of *H. madagascariensis* were cut into small pieces and dried in the shade (24 ± 2°C) for approximately 30 days. The leaves were directly dried under the same conditions. Each of these was ground at ambient temperature, using a mechanical grinder to obtain a fine powder.

#### 2.2.2. Methanol Extracts

The powders obtained were extracted as reported by Foutse et al. [13]. For this purpose, 300 g of each powder was mixed with 1000 mL methanol and maintained at room temperature for 72 h with twice daily homogenization. The obtained solution was then filtered using Whatman No. 1 filter paper. The filtrate was concentrated using a “RE52AA” rotary evaporator at 55°C. These extracts were dried in an oven for 48 h at 40°C to remove residual solvent.

#### 2.2.3. Aqueous Extracts

The powders obtained were extracted according to Atsafack et al.'s protocol [14]. For this purpose, 50 g of each powder was added to 300 mL of distilled water and then boiled at 100°C for 15 min. The mixture was then left to stand for 6 h in the dark and further filtered using Whatman No. 1 filter paper. The filtrate was dried in an oven at 40°C for 48 h to obtain an aqueous crude extract.

#### 2.2.4. Hydroalcoholic Extracts

The extracts were obtained by following the protocol reported by Etame Loé et al. [15]. For this purpose, 100 g powder was added to 300 mL of palm wine and boiled at 100°C for 15 min. After 6 h in the dark at room temperature, the mixture was filtered using Whatman No. 1 filter paper. The filtrate was dried in an oven at 45°C to obtain a crude hydroalcoholic extract.

From each plant material and extraction solvent, the extract was weighed and the yield (%) calculated based on the mass of the sample of plant material. Those extracts were frozen during antibacterial evaluation.

#### 2.2.5. Antibacterial Activity of Plant Extracts

The minimum inhibitory concentrations (MICs) of the extracts were determined by the broth microdilution method [16]. In each of the 96 wells of a microtiter plate, 100 *μ*L of Mueller–Hinton broth was introduced. Subsequently, 100 *μ*L extract prepared at 4096 *μ*g/mL was added to the upper wells. A series of twofold dilutions was carried out to obtain concentrations of extracts ranging from 8 to 1024 *μ*g/mL. Finally, 100 *μ*L of bacterial inoculum at 1.5 × 106 CFU/mL was added to each well. The inoculum was prepared from overnight bacterial cell culture. For that purpose, three to four colonies of each strain were introduced into an assay tube containing 10 mL of sterilised distilled water and adjusted to 0.5 McFarland turbidity scale, that is, approximately 1.5 × 108 CFU/mL. The purity of each strain was verified using specific culture medium prior to antibacterial testing. Ciprofloxacin was used as a positive control at concentrations ranging from 2 to 256 *μ*g/mL, while 2.5% DMSO was used as negative control. The plates were sealed and incubated at 37°C for 24 h. Each test was performed in triplicate and repeated thrice. At the end of the incubation time, the bacterial growth was monitored using p-iodonitrotetrazolium chloride (INT) 2%. For this purpose, 40 *μ*L of the INT solution was introduced into each well. The plates were incubated at 37°C for 30 min, and the MIC value was determined as the smallest concentration of the substance for which no change in INT colour was observed [13]. Based on the MIC values, the activity of the extracts was considered as significant (MIC<100 *μ*g/mL), moderate (100≤ MIC≤ 625 *μ*g/mL), and weak (MIC> 625 *μ*g/mL) [17].

Following MIC determination, minimum bactericidal concentrations (MBCs) were determined as reported by Foutse et al. [13]. In brief, 10 *μ*L aliquots of the medium drawn from wells which did not show any growth during MIC assay were subcultured on Mueller–Hinton agar and incubated for 18 hours at 35°C. The lowest concentration of the substance from which negative growth was recorded was considered as MBC. The MBC/MIC ratio made it possible to distinguish the bactericidal effect (MBC/MIC≤4) and the bacteriostatic effect (MBC/MIC> 4).

## 3. Results and Discussion

### 3.1. Results

#### 3.1.1. Extraction Yields

The extraction yields varied with the part of the plant, the harvest period, and the extraction method/solvent (Table 2). The highest yields were obtained using the bark regardless of the harvest period and extraction solvent. In contrast, the lowest extraction yields were obtained using the roots. Furthermore, methanol extracts from the leaves and bark had the highest extraction yields when harvested in the morning. These yields decreased during the day relatively to leaves and barks. The hydroalcoholic extract had the best yield whatever the plant part and the harvest period.

#### 3.1.2. Antibacterial Activities of Methanol Extracts

*(1) Leaf Extract*. The methanol extracts of the leaves of *H. madagascariensis* were active on 34 out of the 35 strains tested with MICs values ranging between 64 and 1024 *µ*g/mL (Table 3). Their activity was significant on 3 out of the 35 strains tested. The activity of the 6 a.m. extract was significant on 1 strain, *Serratia marcescens*, out of the 35 strains tested and moderate on 18 strains. The activity of the midday extract was significant on 2 out of the 35 strains tested and moderate on 22 strains. The activity of the evening extract was significant on 2 strains and moderate on 29 strains.

These results show that the leaves have better activity when they are harvested in the evening with MICs between 64 and 512 *µ*g/mL. The calculation of the MBC/MIC ratio reveals that the extracts had a bactericidal effect on 14 of the 35 strains tested and a bacteriostatic one on 21 of the 35 strains tested.

*Escherichia coli* and *Staphylococcus aureus* strains were more sensitive to methanol extracts from the leaves. Among the 7 *Escherichia coli* strains tested, one strain presented a significant sensitivity and 5 strains presented a moderate sensitivity. *S. aureus* strains tested showed moderate sensitivity in 6 of the 9 strains tested, including methicillin-resistant strains.

*(2) Bark Extracts*. The methanol extracts of the barks of *H. madagascariensis,* regardless of the time of harvest, were active on all 35 strains tested with MICs between 8 and 1024 *µ*g/mL (Table 4). Their activity was significant on 9 out of the 35 tested strains.

The activity of the morning extract was significant on 4 strains and moderate on 27 strains. The activity of the midday extract was significant on 8 and moderate on 23 strains and, finally, the activity of the evening extract was significant on 9 and moderate on 25 strains. These results show that the barks have better activity when they are harvested in the evening with MICs between 8 and 512 *µ*g/mL.

The MBC/MIC ratio revealed that the extracts had a bactericidal effect on 13 of the 35 strains tested and a bacteriostatic one on 22 strains.

The strains of *S. aureus* and *E. coli* appear to be more sensitive to the methanol extract of barks. Among the 9 strains of *S. aureus*, 6 strains showed a moderate activity and 3 strains a weak activity.

*(3). Root Extract*. The methanol extracts of the roots of *H. madagascariensis*, regardless of the harvesting time, were active on 24 of the 35 strains tested with MICs between 128 and 1024 *µ*g/mL (Table 5). The activity of morning extract was moderate on 15 strains, that of midday on 16, and that in the evening on 20 strains. The MBC/MIC ratio showed that the extracts had a bactericidal effect on 8 strains and a bacteriostatic effect on 27 strains. *S. aureus* strains were more sensitive to extracts from the roots. Indeed, among the 9 strains of *S. aureus*, including the methicillin-resistant strains, 8 strains showed moderate activity.

#### 3.1.3. Antibacterial Activity of Aqueous Extracts

*(1) Leaf Extracts*. The aqueous extracts of the leaves of *H. madagascariensis*, regardless of the time of harvest, were active on 28 out of the 35 strains tested with MICs between 128 and 1024 *µ*g/mL (Table 6). The activity was moderate on 22 strains when harvested in the morning, 4 at midday, and one in the evening. The MBC/MIC ratio showed that the extracts had a bactericidal effect on one strain and a bacteriostatic effect on the other 34.

*(2) Bark Extracts*. The aqueous extracts of the barks of *H. madagascariensis* were active on all the strains tested (35) with MICs between 64 and 1024 *µ*g/mL (Table 7). Their activity was significant on at least 4 strains. The activity was moderate on 21 strains with the morning extract and 8 strains with the midday extract. The activity of the evening extracts was significant on 4 and moderate on 30 strains. The MBC/MIC ratio revealed that the extracts had a bactericidal effect on 6 of the 35 strains tested.

*(3) Roots Extract*. The aqueous extract of the roots of *H. madagascariensis* was found to be almost inactive on the bacterial strains tested.

#### 3.1.4. Antibacterial Activity of Hydroalcoholic Extracts

The palm wine extracts from leaves, bark, and roots of *H. madagascariensis* were found to be inactive on the bacterial strains tested.

## 4. Discussion

Plants are an undeniable source of active substances, many of which are effective at the level of traditional pharmacopoeia as well as at the level of scientific research [17]. Among the multitude of active plants well established all over the word, the unanimity about the part used and the extraction solvents is a permanent quest. Moreover, few studies are interested in the contributions of the agroecological zone, the age, and the daily metabolic variation within the plant. If the standardisation of the active principles of plants is a prerequisite for their efficient use for the pharmacological purpose, it seems certain that it inevitably involves the mastery of the conditions which could affect the variability of the principles at both qualitative and quantitative levels. It is in this context that this work falls, with the general objective being to identify the optimal conditions for the antibacterial activity of *H. madagascariensis* extract.

The extraction yields of *H. madagascariensis* obtained vary from one part of the plant to another and according to the harvest period and extraction solvent used. For methanol extracts, the best yields were obtained using the bark harvested in the morning and at midday. Similarly, the lowest yield was obtained using the roots harvested in the morning (6 a.m.). The variation in extraction yield reflects that of potent antimicrobial compounds whose high concentrations could be obtained at certain times of the day. Similar results have been reported by several authors. Indeed, Ramezani et al. [18] reported a maximum essential oils extract with *Eucalyptus nicholii* and *Rosmarinus officinalis* in the central region of Iran at midday and in the afternoon (6 p.m.). Similarly, Magne [10] reported that the extraction yields of H. *madagascariensis* varied with the plant part and the collection period. These results, therefore, highlight the importance of the plants harvesting period for their pharmacological properties since the yield is an essential component for the pharmacological study of the active substances from plants.

The barks well concentrated the phytochemical constituents of *H. madagascariensis*, whatever the time of harvest. The variation in extraction yield reflects that of potentially antimicrobial compounds whose high concentrations could be obtained with the barks. Thus, the barks of *H. madagascariensis* could be privileged parts of harvest for the pharmacological use of this plant. Similar results were reported by Wang and Liu [19] who found large variation in the composition among oils from roots, fruits, stems, leaves, and flowers of *Litsea cubeba*. These results highlight the importance of plant part for the exploration or exploitation of pharmacological properties.

The different yields of methanol and aqueous extracts of the bark of *H. madagascariensis* obtained at certain times of the day approximate to those obtained by Ebelle Etame et al. [9] and Magne [10], i.e., 14.5% and 19.79%, respectively. These high yields constitute an important factor in the bioavailability of the extracts for later use as a basic principle of phytomedicine.

To make a juxtaposition between the ethnobotanical and scientific means, on the one hand and, and to raise the interest of the solvent, on the other hand, the methanol, aqueous, and hydroalcoholic extracts of the leaves, bark, and roots of *H. madagascariensis* were prepared. From the antibacterial activity of different extracts, as a function of solvent, part, and harvesting period, it appears that the barks are the most active part of the plant, with methanol followed by water being the best extraction solvent. The barks of *H. madagascariensis* could be the best part concentrating compounds responsible for the antibacterial activity. The results are in line with earlier mentioned data where the bark was found to be the suitable plant part for antifungal substances. The antimicrobial activity of plant extracts is due to the presence of secondary metabolites, and the activity of some classes of phytochemicals has been demonstrated [20]. According to previous results on this plant, the activity observed could be associated with the presence of harunganin [21, 22], Malagasy anthrone [23], harongin anthrone [22], harunmadagascarins A and B, kengaquinone, physcion [24], madagascol [25], vismiaquinone [24, 25], betulinic acid, kaempferol-3-O-*β*-D-glucopyranoside and madagascin, ferruginin A [7].

The results of the antibacterial activity corroborate those of Krief [8] who showed that the methanol extract of bark of *H. madagascariensis* had antibacterial properties with MIC values varying between 32 and 1024 *µ*g/mL. Moreover, the result brings to light the need for complementarity between ethnobotanical knowledge and scientific research for more effective development of medicinal plants. In addition, under traditional conditions, it highlights the use of water rather than palm wine in the preparation of the decoction for antibacterial use.

It appeared that extracts are more active when the parts of the plant are harvested in the evening (6 p.m.). The plant uses solar energy for the synthesis of primary metabolites, which are further converted into secondary metabolites, with the concentration of each depending on the plant and several conditions [26]. The accumulation of these compounds responsible for antibacterial activity could be maximum in *H. madagascariensis* (around 6 p.m.). These results corroborate those of Da Silva et al. [27] who found a maximum of bioactive compounds in some plants when harvested in the afternoon. They also corroborate Ramezani et al.'s findings [18]. This result highlights once more the interest in considering endogenous and exogenous factors on which the concentration of the active ingredients in the plant depends. According to the World Health Organisation [28], medicinal plants should be harvested in the optimal season or time to ensure the production of the highest possible quality and quantity of bioproducts.

By comparing the MIC values obtained from the different extraction solvents, regardless of the time of the day and the harvested parts, it appears that methanol is the best extraction solvent with MICs between 8 and 1024 *µ*g/mL. According to Osama [29], during the extraction with a solvent, phytomolecules dissolve in the extraction solvent according to their polarity. Thus, the active compounds of this plant could be a mixture of polar and semipolar compounds best extracted with methanol. These results are in line with those of Benbrinis [30] who found that, compared to water, the methanol extract of the aerial part of *Santolina chamaecyparissus*, a medicinal plant from the pharmacopoeia tradition of Algeria, had a considerable antibacterial effect on the strains tested.

The methanol extracts of bark from *H. madagascariensis* were more active on *E. coli* and *S. aureus* strains which are resistant to some commonly used antibiotics in Cameroon including ampicillin, piperacillin/tazobactam, nalidixic acid, ciprofloxacin, ofloxacin, kanamycin, erythromycin, tetracycline, flomoxef, and chloramphenicol. The efficiency of the methanol extracts of the barks of *H. madagascariensis*, thus, proves that the bark of this plant is a potential source of antibacterial molecules capable of fighting against diseases mostly caused by these microorganisms, including those caused by their multiresistant forms. The results are complementary to previous works, which reported the antibacterial activity of the aqueous extract of the leaves and bark of *H. madagascariensis* on *S. paratyphi*, *S. aureus*, and *E. coli*. [7].

## 5. Conclusion

This study focused on the evaluation of the influence of the plant's parts, the harvest period, and the solvent on the yield and antibacterial activity of *H. madagascariensis*. The aim was to highlight the optimal conditions for antibacterial activity of *H. madagascariensis* extracts. It appeared that the highest yields were obtained with the bark of *H*. *madagascariensis* regardless of the harvest period and the extraction solvent. It also appeared that bark extracts had the best antibacterial activity. The ideal time for harvesting the barks for good antibacterial activity was the evening (6 p.m.), and the solvent that made it possible to concentrate the maximum of the antibacterial compound was methanol. This study further confirms the use of the bark of *H. madagascariensis* as an antibacterial agent and, better still, the use of the barks harvested at 6 p.m. and extracted with methanol.

## Figures and Tables

**Table 1:** Sensitivity and resistance profile of the bacterial strains used for the experiment.

Bacteria characteristics
*Escherichia coli*
ATCC 10536 reference strain
ATCC 8739 reference strain
E.C 99 clinical isolate: IPM^S^, AX^I^, NOR^S^, CFM^S^, CRO^S^, CIP^S^, AN^I^, CHL^S^
E.C 136 clinical isolate: IPM^S^, AX^R^, NOR^S^, CFM^S^, CRO^S^, CIP^S^, AN^S^, CHL^S^
E.C 137 clinical isolate: IPM^S^, AX^S^, NOR^S^, CFM^S^, CRO^S^, CIP^S^, AN^I^, CHL^S^
E.C 2 clinical isolate: TZP^S^, CTX^S^, CAZ^S^, E^S^, IPM^S^, FUR^S^, AMP^R^, TIC^R^, GN^R^, TOB^R^, ANR, NAL^R^, SIP^R^, OFL^R^
E.C 4 clinical isolate: CTX^S^, CAZ^S^, E^S^, IPM^S^, GN^S^, TOB^S^, FUR^S^, AMP^R^, TIC^R^, TZP^R^, NA^R^, CIP^R^, OFX^R^
E.C 5 clinical isolate: TEP^S^, CTX^S^, CAZ^S^, E^S^, IPM^S^, AN^S^, GN^S^, TOB^S^, CIP^S^
*Citrobacter freundii*
CIT B 80 clinical isolate: STX^R^, DO^R^, GN^R^, AMC^R^, TE^R^, CIP^R^, CHL^R^, AK^S^, CT^S^, AX^R^, CRO^R^, CAZ^R^, IPM^S^, NOR^R^, AN^S^
CIT B 81 clinical isolate: STX^S^, DO^I^, GN^S^, AMC^R^, TE^R^, CIP^S^, CHL^S^, AK^S^, CT^S^, AX^R^, CRO^R^, CAZ^S^, IPM^S^, NOR^S^, AN^S^
*Serratia marcescens*
SERB 115 clinical isolate: STX^R^, DO^R^, GN^S^, AMC^S^, TE^R^, CIP^S^, CHL^S^, AK^S^, CT^S^, AX^R^, CRO^S^, CAZ^S^, IPM^S^, NOR^S^, AN^S^
*Yersinia enterocolitica*
YERB 121 clinical isolate: STX^S^, DO^R^, GN^S^, AMC^S^, TE^R^, CIP^S^, CHL^S^, AN^S^, CT^R^, AX^R^, CRO^I^, CAZ^I^, IPM^S^, NOR^S^, AN^S^
YERBI clinical isolate: STX^S^, DO^R^, GN^S^, AMC^R^, TE^R^, CIP^S^, CHL^R^, AK^S^, CT^R^, AX^R^, CRO^I^, CAZ^I^, IPM^S^, NOR^I^, AN^S^
*Enterobacter aerogenes*
ENT 51 clinical isolate: STX^R^, DO^R^, GN^S^, AMC^I^, TE^R^, CIP^S^, CHL^S^, AK^S^, CT^R^, AX^R^, CRO^S^, CAZ^I^, IPM^S^, NOR^S^, AN^I^
ENT 119 clinical isolate: IPM^S^, AX^I^, NOR^S^, CFM^S^, CRO^S^, CIP^S^, AN^I^, CHL^S^
ENT 167 clinical isolate: STX^R^, DO^R^, GN^S^, AMC^R^, TE^R^, CIP^I^, CHL^S^, AK^S^, CT^R^, AX^R^, CRO^I^, CAZ^I^, IPM^S^, NOR^R^, AN^R^
*Klebsiella pneumoniae*
KL 111 clinical isolate: IPM^S^, AX^I^, NOR^S^, CFM^S^, CRO^I^, CIP^S^, AN^I^, CHL^S^
KLPB101 clinical isolate: STX^S^, DO^S^, GN^S^, AMC^I^, TE^S^, CIP^S^, CHL^S^, AK^S^, CT^S^, AX^I^, CRO^I^, CAZ^S^, IPM^S^, NOR^S^, AN^S^
*Salmonella enterica* serovar *typhi*
ATCC 6539 reference strain
SAL 9 clinical isolate: IPM^S^, AX^R^, NOR^S^, CFM^S^, CRO^R^, CIP^S^, AN^I^, CHL^I^
*Sal* clinical isolate: AM^I^, IPM^S^, AX^S^, NOR^S^, AN^I^, CFM^S^, CRO^R^, CIP^S^, AN^I^, CHL^S^
*Salmonella paratyphi* B clinical isolate: AM^R^, TE^R^, SXT^R^, AN^R^, CIP^S^, CHL^S^
*Salmonella typhimurium* clinical isolate: AM^R^, TE^R^, SXT^R^, NA^R^, CIP^S^, CHL^S^
*Pseudomonas aeruginosa*
ATCC 27853 reference strain
*Providencia stuartii*
PS NEA 16 clinical isolate MDR: protein synthesis inhibitor OmpF of OmpC
Bacteria Gram-positive
*Staphylococcus aureus*
STAPH 1 clinical isolate: IPM^S^, DO^S^, AMC^S^, E^S^, VA^S^
MRSA 9 resistant clinical isolate: OFX^R^, FLX^R^, K^R^, E^R^, CHL^R^, IMP/CS^R^
MRSA 11 resistant clinical isolate: OFX^R^, K^R^, E^R^, CIP^R^, IM/CS^R^
MRSA 12 resistant clinical isolate: OFX^R^, FLX^R^, K^R^, E^R^, IM/CS^R^
MRSA 3 resistant clinical isolate: OFX^R^, K^R^, E^R^, TE^R^
3 clinical isolate: IPM^S^, AMC^S^, AM^S^, DO^S^, VA^S^, E^S^
ST 120 clinical isolate: IPM^S^, AX^S^, AM^S^, DO^S^, VA^S^, E^S^
SA 3 clinical isolate: CC^R^, QUI^S^/DAL^S^, LIN^S^, VA^R^, TE^R^, TIG^S^, NIT^S^, RIF^R^, TRI^R^/SUL^R^
*Streptococcus* sp.
STR 7 clinical isolate: CC^S^, QUI^S^, LIN^s^, VA^S^, TE^R^, TIG^S^, NIT^S^, STX^R^

AK: amikacin; AM: ampicillin; CHL: chloramphenicol; CFM: cefixime; CIP: ciprofloxacin; CRO: ceftriaxone; DO: doxycycline; E: erythromycin; FLX: flomoxef; IM/CS: imipenem/sodium cilastatin; K: kanamycin; NA: nalidixic acid; NOR: norfloxacin; OFX: ofloxacin; TE: tetracycline; STX: cotrimoxazole; AMC: amoxicillin + clavulanic acid; AX: amoxicillin; AZM: azithromycin; CAZ: ceftazidim; CC: clindamycin; CN: cefalexin; CT: ceftolozane + tazobactam; IPM: imipenem; L: lincomycin; NOR: norfloxacin; TE: tetracycline; QUI: quinupristin; LIN: linezolid; TIG: tigecycline; NIT: nitrofurantoin; RIF: rifampicin; VA: vancomycin; GN: gentamicin; TOB: tobramycin; TIC: ticarcillin; TZP: piperacillin/tazobactam; CTX: cefotaxime; R: resistant; S: sensitive; I: intermediate.

**Table 2:** Extraction yield (%) of methanol, aqueous, and palm wine decoctions from *H. madagascariensis* at different harvest times/periods.

Parts	Solvents								
	Methanol			Palm wine			Water		
	6 a.m.	12 p.m.	6 p.m.	6 a.m.	12 p.m.	6 p.m.	6 a.m.	12 p.m.	6 p.m.
Leaves	11.69	4.77	7.39	26.57	24.53	24.48	7.48	0.94	0.94
Bark	15.47	15.02	13.36	28.36	24.05	26.95	26.34	25.2	2.02
Roots	4.3	6.59	9.55	22.43	26.72	24.82	4.5	1.7	1.98

**Table 3:** Minimum inhibitory concentrations (MICs) and minimum bactericidal concentrations (MBCs) of methanol extracts from the leaves of *H. madagascariensis* (*µ*g/mL).

		Leaves									CIP		
				6 a.m.			12 p.m.			6 p.m.		
		MIC	MBC	R	MIC	MBC	R	MIC	MBC	R	MIC	MBC	R
Gram-negative bacteria													
*Escherichia coli*	ATCC 8739	256	—	Nd	256	—	Nd	256	1024	4	≤0.5	8	16
	ATCC 10536	512	—	Nd	512	—	Nd	256	1024	4	2	64	8
	EC 5	128	—	Nd	64	—	Nd	64	1024	16	4	8	2
	EC 4	512	—	Nd	256	512	2	128	512	4	≤0.5	64	128
	EC 2	512	—	Nd	512	1024	2	512	—	Nd	4	32	8
	EC 136	1024	—	Nd	1024	—	Nd	256	—	Nd	4	64	16
	EC 137	—	—	—	1024	—	Nd	512	—	Nd	4	32	8
*Citrobacter freundii*	CITB 80	—	—	—	—	—	—	256	—	Nd	1	32	32
	CITB 81	—	—	—	256	1024	4	64	1024	16	1	64	64
*Serratia marcescens*	SERB 115	64	128	2	64	128	2	128	1024	8	1	32	32
*Yersinia enterocolitica*	YERB 121	1024	—	Nd	128	1024	8	128	—	Nd	≤0.5	/4	8
	YERBI	—	—	—	512	1024	2	128	—	Nd	1	2	2
*Salmonella enterica* serovar *typhi*	ATCC 6539	128	1024	8	128	1024	8	128	—	Nd	4	8	2
	SAL 9	—	—	—	256	—	Nd	256	—	Nd	4	8	2
	S AL	128	—	Nd	128	—	Nd	128	—	Nd	0.5	64	128
*Salmonella paratyphi* B		256	—	Nd	256	—	Nd	—	—	—	2	16	8
*Salmonella typhimurium*		256	—	Nd	256	1024	4	512	—	Nd	4	32	8
*Pseudomonas aeruginosa*	ATCC 27853	—	—	—	512	1024	2	256	—	Nd	16	32	2
	PA 16	512	—	Nd	1024	—	Nd	512	—	Nd	2	32	16
*Providencia stuartii*	PS NEA 16	—	—	—	—	—	—	—	—	—	2	16	8
*Klebsiella pneumoniae*	KL 111	1024	—	Nd	—	—	—	256	—	Nd	32	64	2
	KLPB101	—	—	—	—	—	—	—	—	—	8	32	4
*Enterobacter aerogenes*	ENT 119	—	—	—	128	1024	8	256	1024	4	1	4	4
	ENT 167	256	1024	4	512	1024	2	256	—	Nd	16	32	2
	ENT 51	—	—	—	1024	—	Nd	512	—	Nd	8	16	2
Gram-positive bacteria													
*Staphylococcus aureus*	STAPH 1	256	—	Nd	256	—	Nd	128	1024	8	16	32	2
	3	1024	—	Nd	256	—	Nd	256	—	Nd	1	8	8
	MRSA 3	256	1024	4	512	1024	2	512	1024	2	0.5	2	4
	MRSA 9	256	1024	4	128	—	Nd	128	—	Nd	0.5	32	64
	MRSA 12	512	1024	2	512	1024	2	512	1024	2	8	32	4
	MRSA 13	512	—	Nd	512	—	Nd	512	—	Nd	1	16	16
	ST 120	—	—	—	—	—	—	512	—	Nd	2	32	16
	ST 113	1024	1024	1	—	—	—	—	—	—	4	32	8
	SA 3	128	1024	8	128	—	Nd	128	1024	8	1	8	8
*Streptococcus* sp.	STR 7	512	—	Nd	1024	—	Nd	256	1024	4	1	64	64

MIC: minimum inhibitory concentration; MBC: minimum bactericidal concentration; R: MBC/MIC; CIP: ciprofloxacin; Nd: not determined; —: no activity.

**Table 4:** Minimum inhibitory concentrations (MICs) and minimum bactericidal concentrations (MBCs) of methanol extracts from the bark of *H. madagascariensis* (*µ*g/mL).

		Bark									CIP		
				6 a.m.			12 p.m.			6 p.m.		
		MIC	MBC	R	MIC	MBC	R	MIC	MBC	R	MIC	MBC	R
Gram-negative bacteria													
*Escherichia coli*	ATCC 8739	128	1024	8	64	—	Nd	64	1024	16	≤0.5	8	16
	ATCC 10536	256	1024	4	128	—	Nd	128	512	4	2	64	8
	EC 5	256	—	Nd	256	1024	4	128	—	Nd	4	8	2
	EC 4	128	256	2	256	—	Nd	128	1024	8	≤0.5	64	128
	EC 2	512	—	Nd	1024	—	Nd	512	—	Nd	4	32	8
	EC 136	512	—	Nd	512	—	Nd	512	1024	2	4	64	16
	EC 137	256	512	2	256	1024	4	256	1024	4	4	32	8
*Citrobacter freundii*	CITB 80	128	1024	8	128	—	Nd	64	512	8	1	32	32
	CITB 81	256	512	2	256	1024	4	256	512	2	1	64	64
*Serratia marcescens*	SERB 115	64	512	8	64	512	8	64	256	4	1	32	32
*Yersinia enterocolitica*	YERB 121	256	—	Nd	32	—	Nd	32	1024	32	≤0.5	/4	8
	YERBI	512	1024	2	1024	—	Nd	256	1024	4	1	2	2
*Salmonella enterica* serovar *typhi*	ATCC 6539	128	—	Nd	32	—	Nd	32	1024	32	4	8	2
	SAL 9	256	—	Nd	128	—	Nd	128	—	Nd	4	8	2
	S AL	128	—	Nd	128	1024	8	64	1024	16	0.5	64	128
*Salmonella paratyphi* B		128	128	1	256	1024	4	256	512	2	2	16	8
*Salmonella typhimurium*		512	—	Nd	512	—	Nd	128	—	Nd	4	32	8
*Pseudomonas aeruginosa*	ATCC 27853	512	—	Nd	512	1024	2	256	512	2	16	32	2
	PA 16	512	—	Nd	512	—	Nd	256	—	Nd	2	32	16
*Providencia stuartii*	PS NEA 16	512	—	Nd	256	—	Nd	256	—	Nd	2	16	8
*Klebsiella pneumoniae*	KL 111	256	—	Nd	512	—	Nd	256	1024	4	32	64	2
	KLPB101	—	—	—	—	—	—	256	1024	4	8	32	4
*Enterobacter aerogenes*	ENT 119	128	—	Nd	128	—	Nd	128	—	Nd	1	4	4
	ENT 167	256	—	Nd	128	1024	8	256	1024	4	16	32	2
	ENT 51	1024	—	Nd	512	—	Nd	1024	—	Nd	8	16	2
Gram-positive bacteria													
*Staphylococcus aureus*	STAPH 1	64	256	4	64	512		64	512	8	16	32	2
	3	64	—	Nd	≤8	—	Nd	32	—	Nd	1	8	8
	MRSA 3	512	—	Nd	512	1024	2	256	512	2	0.5	2	4
	MRSA 9	64	—	Nd	8	1024	128	8	—	Nd	0.5	32	64
	MRSA 12	256	1024	4	128	1024	8	128	1024	8	8	32	4
	MRSA 13	512	—	Nd	512	—	Nd	256	—	Nd	1	16	16
	ST 120	—	—	—	1024	—	Nd	256	—	Nd	2	32	16
	ST 113	—	—	—	512	—	Nd	512	—	Nd	4	32	8
	SA 3	128	1024	8	64	64	1	128	—	Nd	1	8	8
*Streptococcus* sp.	STR 7	512	1024	2	256	—	Nd	128	512	4	1	64	64

MIC: minimum inhibitory concentration; MBC: minimum bactericidal concentration; R: MBC/MIC; CIP: ciprofloxacin; Nd: not determined; —: no activity.

**Table 5:** Minimum inhibitory concentrations (MICs) and minimum bactericidal concentrations (MBCs) of methanol extracts from the roots of *H. madagascariensis* (*µ*g/mL).

		Roots									CIP		
				6 a.m.			12 p.m.			6 p.m.		
		MIC	MBC	R	MIC	MBC	R	MIC	MBC	R	MIC	MBC	R
Gram-negative bacteria													
*Escherichia coli*	ATCC 8739	512	—	Nd	512	—	Nd	256	—	Nd	≤0.5	8	16
	ATCC 10536	—	—	—	—	—	—	—	—	—	2	64	8
	EC 5	512	—	Nd	512	—	Nd	256	—	Nd	4	8	2
	EC 4	—	—	—	—	—	—	—	—	—	≤0.5	64	128
	EC 2	—	—	—	—	—	—	—	—	—	4	32	8
	EC 136	—	—		—	—	—	—	—	—	4	64	16
	EC 137	—	—	—	—	—	—	—	—	—	4	32	8
*Citrobacter freundii*	CITB 80	—	—	—	—	—	—	1024	—	Nd	1	32	32
	CITB 81	—	—	—	—	—	—	—	—	—	1	64	64
*Serratia marcescens*	SERB 115	128	1024	8	256	—	Nd	128	—	Nd	1	32	32
*Yersinia enterocolitica*	YERB 121	1024	—	Nd	128	—	Nd	512	512	1	≤0.5	/4	8
	YERBI	—	—	—	—	—	—	—	—	—	1	2	2
*Salmonella enterica* serovar *typhi*	ATCC 6539	256	1024	4	512	—	Nd	256	1024	4	4	8	2
	SAL 9	1024	—	Nd	1024	—	Nd	1024	—	Nd	4	8	2
	S AL	128	256	2	256	1024	4	256	1024	4	≤0.5	64	128
*Salmonella paratyphi* B		128	—	Nd	512	—	Nd	256	—	Nd	2	16	8
*Salmonella typhimurium*		—	—	—	—	—	—	512	1024	2	4	32	8
*Pseudomonas aeruginosa*	ATCC 27853	1024	—	Nd	—	—	—	—	—	—	16	32	2
	PA 16	128	—	Nd	—	—	—	128	—	Nd	2	32	16
*Providencia stuartii*	PS NEA 16	—	—	—	—	—	—	1024	—	Nd	2	16	8
*Klebsiella pneumoniae*	KL 111	—	—	—	—	—	—	—	—	—	32	64	2
	KLPB101	—	—	—	—		—	—	—	—	8	32	4
*Enterobacter aerogenes*	ENT 119	128	—	Nd	512	—	Nd	512	—	Nd	1	4	4
	ENT 167	1024	1024	1	1024	1024	1	512	1024	2	16	32	2
	ENT 51	—	—	—	—	—	—	—	—	—	8	16	2
Gram-positive bacteria													
*Staphylococcus aureus*	STAPH 1	512	1024		512	—	Nd	512	—	Nd	16	32	2
	3	1024	—	Nd	512	—	Nd	512	—	Nd	1	8	8
	MRSA 3	512	—	Nd	512	1024	2	512	1024	2	0.5	2	4
	MRSA 9	256	—	Nd	256	—	Nd	128	1024	8	0.5	32	64
	MRSA 12	128	1024	8	256	—	Nd	256	1024	4	8	32	4
	MRSA 13	128	—	Nd	512	—	Nd	512	—	Nd	1	16	16
	ST 120	—	—	—	—	—	—	—	—	—	2	32	16
	ST 113	512	—	Nd	512	—	—	512	1024	2	4	32	8
	SA 3	256	—	Nd	512	1024	2	256	—	Nd	1	8	8
*Streptococcus* sp.	STR 7	—	—	—	—	—	—	512	—	Nd	1	64	64

MIC: minimum inhibitory concentration; MBC: minimum bactericidal concentration; R: MBC/IMC; CIP: ciprofloxacin; Nd: not determined; —: no activity.

**Table 6:** Minimum inhibitory concentrations (MICs) and minimum bactericidal concentrations (MBCs) of the aqueous extracts of the leaves of *H. madagascariensis* (*µ*g/mL).

		Leaves									CIP		
				6 a.m.			12 p.m.			6 p.m.		
		MIC	MBC	R	MIC	MBC	R	MIC	MBC	R	MIC	MBC	R
Gram-negative bacteria													
*Escherichia coli*	ATCC 8739	256	—	Nd	128	—	Nd	512	—	Nd	1	16	16
	ATCC 10536	256	—	Nd	—	—	Nd	—	—	—	2	64	32
	EC 5	256	—	Nd	—	—	Nd	1024	1024	1	4	32	8
	EC 4	512	—	Nd	1024	—	Nd	—	—	—	1	64	64
	EC 2	512	—	Nd	—	—	Nd	1024	—	Nd	4	32	8
	EC 136	512	—	Nd	1024	—	Nd	1024	—	Nd	4	64	16
	EC 137	512	—	Nd	1024	—	Nd	—	—	—	4	32	8
*Citrobacter freundii*	CITB 80	512	—	Nd	1024	—	Nd	—	—	—	1	32	32
	CITB 81	512	—	Nd	1024	—	Nd	—	—	—	1	64	64
*Serratia marcescens*	SERB 115	—	—	—	—	—	—	—	—	—	1	32	32
*Yersinia enterocolitica*	YERB 121	—	—	—	—	—	—	—	—	—	1	4	4
	YERBI	512	—	Nd	512	—	Nd	1024	—	Nd	1	2	2
*Salmonella enterica* serovar *typhi*	ATCC 6539	—	—	—	—	—	—	—	—	—	4	8	2
	SAL 9	512	—	Nd	—	—	—	—	—	—	4	8	2
	S AL	256	256	1	—	—	—	1024	1024	1	0.5	64	128
*Salmonella paratyphi* B		1024	1024	1	—	—	—	—	—	—	2	16	8
*Salmonella typhimurium*		512	—	—	—	—	—	—	—	—	4	4	1
*Pseudomonas aeruginosa*	ATCC 27853	512	—	Nd	1024	—	Nd	1024	—	Nd	16	32	2
	PA 16	256	—	Nd	1024	—	Nd	—	—	—	2	32	16
*Providencia stuartii*	PS NEA 16	—	—	—	—	—	—	—	—	—	2	16	8
*Klebsiella pneumoniae*	KL 111	512	—	Nd	512	—	Nd	1024	—	Nd	32	64	2
	KLPB101	512	—	Nd	—	—	—	1024	—	Nd	8	32	4
*Enterobacter aerogenes*	ENT 119	512	—	Nd	—	—	—	—	—	—	1	4	4
	ENT 167	512	—	Nd	—	—	—	—	—	—	16	32	2
	ENT 51	1024	—	Nd	1024	—	Nd	1024	—	Nd	8	16	2
Gram-positive bacteria													
*Staphylococcus aureus*	STAPH 1	1024	1024	1	—	—	—	—	—	—	16	32	2
	3	—	—	—	—	—	—	—	—	—	1	8	8
	MRSA 3	—	—	—	—	—	—	—	—	—	0.5	2	4
	MRSA 9	128	1024	8	256	—	Nd	1024	1024	1	0.5	32	64
	MRSA 12	1024	1024	1	—	—	—	—	—	—	8	32	4
	MRSA 13	—	—	—	—	—	—	—	—	—	1	16	16
	ST 120	512	—	Nd	—	—	—	—	—	—	2	32	16
	ST 113	1024	1024	1	1024	1024	1	—	—	—	4	32	8
	SA 3	1024	1024	1	—	—	—	—	—	—	1	8	8
*Streptococcus* sp.	STR 7	512	—	—	—	—	—	—	—	—	1	64	64

MIC: minimum inhibitory concentration; MBC: minimum bactericidal concentration: R: MBC/MIC; CIP: ciprofloxacin; Nd: not determined; —: no activity.

**Table 7:** Minimum inhibitory concentrations (MICs) and minimum bactericidal concentrations (MBCs) of the aqueous extracts of the bark of *H. madagascariensis* (*µ*g/mL).

		Bark									CIP		
				6 a.m.			12 p.m.			6 p.m.		
		MIC	MBC	R	MIC	MBC	R	MIC	MBC	R	MIC	MBC	R
Gram-negative bacteria													
*Escherichia coli*	ATCC 8739	—	—	—	—	—	—	64	—	Nd	1	16	16
	ATCC 10536	256	—	Nd	—	—	—	512	—	Nd	2	64	32
	EC 5	—	—	—	—	—	—	512	—	Nd	4	32	8
	EC 4	1024	—	Nd	—	—	—	512	—	Nd	1	64	64
	EC 2	1024	—	Nd	—	—	—	512	—	Nd	4	32	8
	EC 136	512	—	Nd	256	—	Nd	512	—	Nd	4	64	16
	EC 137	512	—	Nd	1024	—	Nd	256	—	Nd	4	32	8
*Citrobacter freundii*	CITB 80	512	—	Nd	1024	—	Nd	256	—	Nd	1	32	32
	CITB 81	—	—	—	—	—	—	512	—	Nd	1	64	64
*Serratia marcescens*	SERB 115	512	—	Nd	512	—	Nd	64	1024	16	1	32	32
*Yersinia enterocolitica*	YERB 121	512	—	Nd	1024	1024	1	128	512	4	1	4	4
	YERBI	512	—	Nd	—	—	—	256	—	Nd	1	2	2
*Salmonella enterica* serovar *typhi*	ATCC 6539	1024	1024	1	1024	1024	1	256	512	2	4	8	2
	SAL 9	1024	1024	1	1024	1024	1	512	—	Nd	4	8	2
	S AL	512	—	Nd	1024	1024	1	128	1024	8	0.5	64	128
*Salmonella paratyphi* B		512	—	Nd	1024	1024	1	512	1024	1	2	16	8
*Salmonella typhimurium*		—	—	—	—	—	—	512	—	Nd	4	4	1
*Pseudomonas aeruginosa*	ATCC 27853	512	—	Nd	—	—	—	1024	—	Nd	16	32	2
	PA 16	256	—	Nd	1024	—	Nd	512	—	Nd	2	32	16
*Providencia stuartii*	PS NEA 16	512	—	Nd	512	—	Nd	64	1024	16	2	16	8
*Klebsiella pneumoniae*	KL 111	256	—	Nd	256	—	Nd	256	—	Nd	32	64	2
	KLPB101	1024	—	Nd	—	—	—	512	—	Nd	8	32	4
*Enterobacter aerogenes*	ENT 119	512	512	1	512	1024	2	128	—	Nd	1	4	4
	ENT 167	1024	1024	1	1024	1024	1	256	1024	4	16	32	2
	ENT 51	256	—	Nd	—	—	—	512	—	Nd	8	16	2
Gram-positive bacteria													
*Staphylococcus aureus*	STAPH 1	512	—	Nd	1024	1024	1	128	512	4	16	32	2
	3	1024	1024	1	512	1024	2	256	1024	4	1	8	8
	MRSA 3	1024	1024	1	1024	1024	1	512	—	Nd	0.5	2	4
	MRSA 9	—	—	—	—	—	—	64	1024	16	0.5	32	64
	MRSA 12	256	—	Nd	512	—	Nd	128	1024	8	8	32	4
	MRSA 13	512	512	1	1024	1024	1	128	1024	8	1	16	16
	ST 120	256	—	Nd	256	—	Nd	256	—	Nd	2	32	16
	ST 113	—	—	—	—	—	—	512	—	Nd	4	32	8
	SA 3	512	—	—	1024	1024	1	256	—	Nd	1	8	8
*Streptococcus* sp.	STR 7	256	—	Nd	—	—	—	256	—	Nd	1	64	64

MIC: minimum inhibitory concentration; MBC: minimum bactericidal concentration; R: MBC/MIC; CIP: ciprofloxacin; Nd: not determined; —: no activity.

## Data Availability

The data supporting the findings of this study are available within the article.
